# Compound Heterozygosity for Y Box Proteins Causes Sterility Due to Loss of Translational Repression

**DOI:** 10.1371/journal.pgen.1005690

**Published:** 2015-12-08

**Authors:** Elizabeth Snyder, Ramani Soundararajan, Manju Sharma, Andrea Dearth, Benjamin Smith, Robert E. Braun

**Affiliations:** The Jackson Laboratory, Bar Harbor, Maine, United States of America; University of Nevada School of Medicine, UNITED STATES

## Abstract

The Y-box proteins YBX2 and YBX3 bind RNA and DNA and are required for metazoan development and fertility. However, possible functional redundancy between YBX2 and YBX3 has prevented elucidation of their molecular function as RNA masking proteins and identification of their target RNAs. To investigate possible functional redundancy between YBX2 and YBX3, we attempted to construct *Ybx2* ^*-/-*^ *;Ybx3* ^*-/-*^ double mutants using a previously reported *Ybx2* ^*-/-*^ model and a newly generated global *Ybx3* ^*-/-*^ model. Loss of YBX3 resulted in reduced male fertility and defects in spermatid differentiation. However, homozygous double mutants could not be generated as haploinsufficiency of both *Ybx2* and *Ybx3* caused sterility characterized by extensive defects in spermatid differentiation. RNA sequence analysis of mRNP and polysome occupancy in single and compound *Ybx2/3* heterozygotes revealed loss of translational repression almost exclusively in the compound *Ybx2/3* heterozygotes. RNAseq analysis also demonstrated that Y-box protein dose-dependent loss of translational regulation was inversely correlated with the presence of a Y box recognition target sequence, suggesting that Y box proteins bind RNA hierarchically to modulate translation in a range of targets.

## Author Summary

The Y-box proteins are evolutionary conserved across eukaryotes. This study focused on two Y-box proteins, YBX2 and YBX3, expressed in testis and known be important for male fertility. Previous studies in male germ cells link YBX2 and YBX3 proteins to RNA masking, however, whether they function in translational repression or mRNA stability during spermatogenesis has not been resolved. *Ybx2*-null mice are known to be infertile due to post-meiotic spermatid defects. To assess the functional role of YBX3 during spermatogenesis, we generated *Ybx3*-null mice. These mice displayed reduced fertility and spermatid differentiation defects. To test if YBX2 and YBX3 are functionally redundant, we attempted to generate double knockout mice. Double mutants could not be generated due to unexpected infertility in the compound *Ybx2/3* heterozygotes. Compound heterozygotes displayed multiple sperm defects indicative of failed post-meiotic germ cell differentiation. Analysis of translational repression in compound *Ybx2/3* heterozygous testes demonstrated a loss of translation repression in mRNAs lacking the Y box recognition sequence. These findings suggest YBX2 and YBX3 function to repress translation through both sequence-specific and non-specific mechanisms in a hierarchical manner.

## Introduction

Post-transcriptional control is critical for gene regulation during spermatogenesis as the majority of germ cell transcription ceases many days prior to the completion of differentiation. Germ cell differentiation (from mitotic to meiotic to post-meiotic) occurs in a step-wise manner and gives rise to distinct morphological arrangements of male germ cells (stages), making the testis a particularly tractable system for investigating post-transcriptional regulation. Because the precise differentiation state of a given germ cell can be accurately determined by its association with other germ cells (the stage), defects in temporal post-transcriptional control can be readily detected. This is particularly useful in post-meiotic germ cells, a population comprised of round spermatids that further differentiate to form elongated spermatids. A large number of transcripts required for post-meiotic germ cell differentiation are first transcribed in round spermatids wherein they are sequestered in translationally repressed cytoplasmic messenger ribonucleoprotein (mRNP) particles for up to 7 days [1,2]. Translational activation in elongated spermatids coincides with spermatid differentiation and impacts several fundamental differentiation processes, including chromatin compaction and flagellar development [3,4]. While many transcripts have been identified as being under post-transcriptional control, the global and transcript-specific mechanisms underlying this control are not yet elucidated.

Several post-transcriptional control regulators have been identified based on their capacity to associate with the 3’ UTRs of *Prm1 and Prm2*, well-characterized transcripts under post-transcriptional control that are required for chromatin compaction in post-meiotic germ cells. Among these include two members of the murine Y-box protein family [5,6]. Y-box proteins are evolutionary conserved nucleic acid binding proteins with distinct, conserved structures found from insects to higher vertebrates [7]. They were first identified based on their ability to bind Y-box DNA elements present in the promoters of eukaryotic genes [8]. Due to a conserved cold-shock domain and four vertebrate-specific basic/aromatic islands, Y-box proteins bind both single- and double-stranded DNA as well as RNA. Additionally, these proteins appear to play compartment-specific roles in DNA and RNA biology. In the nucleus, Y-box proteins are implicated in transcriptional regulation, splicing, DNA repair and transport, while in the cytoplasm they associate with ribonucleoprotein (RNP) complexes and have been referred to as masking proteins and proposed to function as mRNA stabilizers and translational repressors [9].

In the mouse, three Y-box protein-encoding genes have been identified: *Ybx1* (previously known as *Msy1*), *Ybx2* (previously known as *Msy2*), and *Ybx3* (previously known as *Msy4*). Two of the three Y-box genes, *Ybx1* and *Ybx3*, are widely expressed in the developing embryo [10], while all three are expressed in the developing and adult testis [6,10,11]. For all three proteins, the majority of reports implicate them in translational regulation via direct interaction with target RNAs, and in vitro studies suggest they can bind RNA in both a sequence-dependent and independent manner. Both YBX2 and YBX3 preferentially bind an RNA Y-box protein recognition sequence (YRS) ([UAC][CA]CA[UC]C[ACU]) *in vitro*, a sequence observed in many translationally-regulated messages including *Prm1* [12]. Previous studies have shown that in a non-native context the *Prm1* YRS can confer translational repression to a reporter mRNA *in vivo* [13]. These findings suggest that YBX2, YBX3, or a combination of both act on translationally regulated targets via YRS elements.

Based on molecular observations and phenotypic analysis of transgenic models, translational repression appears to be the key function of YBX2 and YBX3 function in post-meiotic germ cells. Both YBX2 and YBX3, which is expressed as a long and short isoform [14], are found in the germ-cell populations in which maximal translational repression occurs and both are components of testis mRNPs that contain repressed *Prm1* mRNA [6]. Loss of YBX2 results in male infertility [15] and a shift in association of some transcripts normally within mRNPs to polysomes [16], while extending the temporal expression of YBX3 during spermatogenesis in transgenic mice inhibits translation of several translationally regulated genes (including *Prm1* and *Prm2*), resulting in dominant male sterility [4].

The overlapping cell-specific expression and similar functionality of YBX2 and YBX3 suggest possible redundancy or cooperativity in post-meiotic germ cells. A similar mechanism has been proposed for YBX1 and YBX3 in the developing embryo [10]. Both YBX1 and YBX3 are expressed during embryogenesis and while *Ybx1* deficient embryos have a late embryonic (E18.5) to early neonatal lethality phenotype, double *Ybx1/Ybx3* mutants have a much earlier (E8.5 to E11.5) embryonic lethality.

In these studies we demonstrate that compound, but not single, heterozygosity for *Ybx2* and *Ybx3* causes male sterility and loss of translational repression, suggesting functional redundancy between *Ybx2* and *Ybx3*.

## Results

### Loss of YBX3 results in sperm defects and sub-fertility

**Fig 1:**
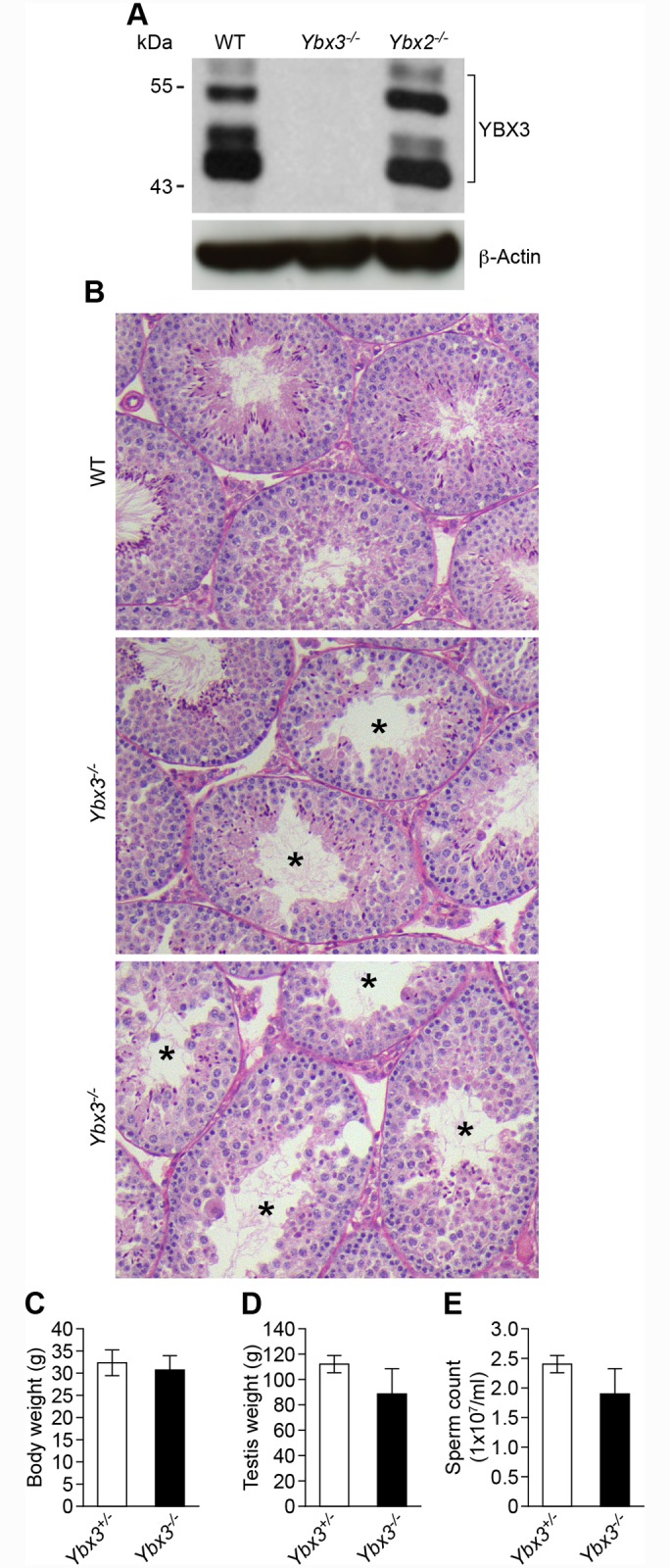
Phenotypic characterization of *Ybx3* ^-/-^ mutant mice. (A) Western blot analysis demonstrating lack of YBX3 protein in *Ybx3* ^-/-^ mutant testis but not *Ybx2* ^*-/-*^ mutant testes. β-actin was used as a loading control. (B) Periodic acid–Schiff–stained sections of adult wildtype and *Ybx3* ^-/-^ mutant testes. Tubule vacuolization (indicated with asterisks) varied from moderate to severe and was dependent on the individual. Testes from two *Ybx3* ^-/-^ mutants are shown for comparison. (C) Body weight, (D) testis weight and (E) epididymal sperm count of adult *Ybx3* ^-/-^ mutants is shown. Values are mean ± SD, N = 6.

To study the impact of *Ybx3* loss on spermatogenesis, we generated a *Ybx3-*null allele (*Ybx3* ^*tm1Reb*^, referred to herein as *Ybx3* ^*-*^) via homologous recombination (S1 Fig). Homozygosity of this allele resulted in a complete loss of both the long (~55 kDa) and short (~43 kDa) YBX3 protein isoforms as detected by western blotting (Fig 1A). *Ybx3-*null testes showed variable phenotypes, with the majority having moderate tubule vacuolization and fewer post-meiotic germ cells than wildtype (Fig 1B). A few animals had severely impacted spermatogenesis with extensive tubule vacuolization and no observable sperm in the epididymis. As a population, *Ybx3-*null males had unchanged body weight, testis weight, and epididymal sperm concentration (Fig 1C–1E). The defects observed in our model are similar, though in some cases slightly more severe, to another YBX3 loss-of function model [10].

### Loss of YBX3 does not impact localization of YBX2

As both YBX2 and YBX3 are thought to perform similar functions and are present in the same cell types, we examined whether the loss of one impacts the production or localization of the other. By western blot, we observed similar YBX3 levels in *Ybx2* null (*Ybx2* ^*tm1Nbh/ tm1Nbh*^, previously reported [15] and hereafter referred to as *Ybx2* ^*-*^) testis relative to control (Fig 1A). To assess if the loss of YBX3 impacts the localization of YBX2 or vice versa, we examined YBX2 and YBX3 by immunofluorescence in *Ybx3* and *Ybx2* null testes, respectively (S2 Fig). YBX2 protein was detected in the cytoplasm of late pachytene spermatocytes and round spermatids, lower in elongating spermatids and absent from late elongated spermatids in control mice and in *Ybx3* mutants, but was absent in *Ybx2* null testis. Conversely, YBX3 was first observed in late pachtyene spermatocytes and was present through early elongated spermatids in control and *Ybx2* ^-/-^ testis but was not detected in *Ybx3* null testis. We conclude that neither protein abundance nor localization of YBX2 or YBX3 is impacted by loss of the other.

### Compound heterozygosity of *Ybx2* and *Ybx3* causes male sterility

**Fig 2:**
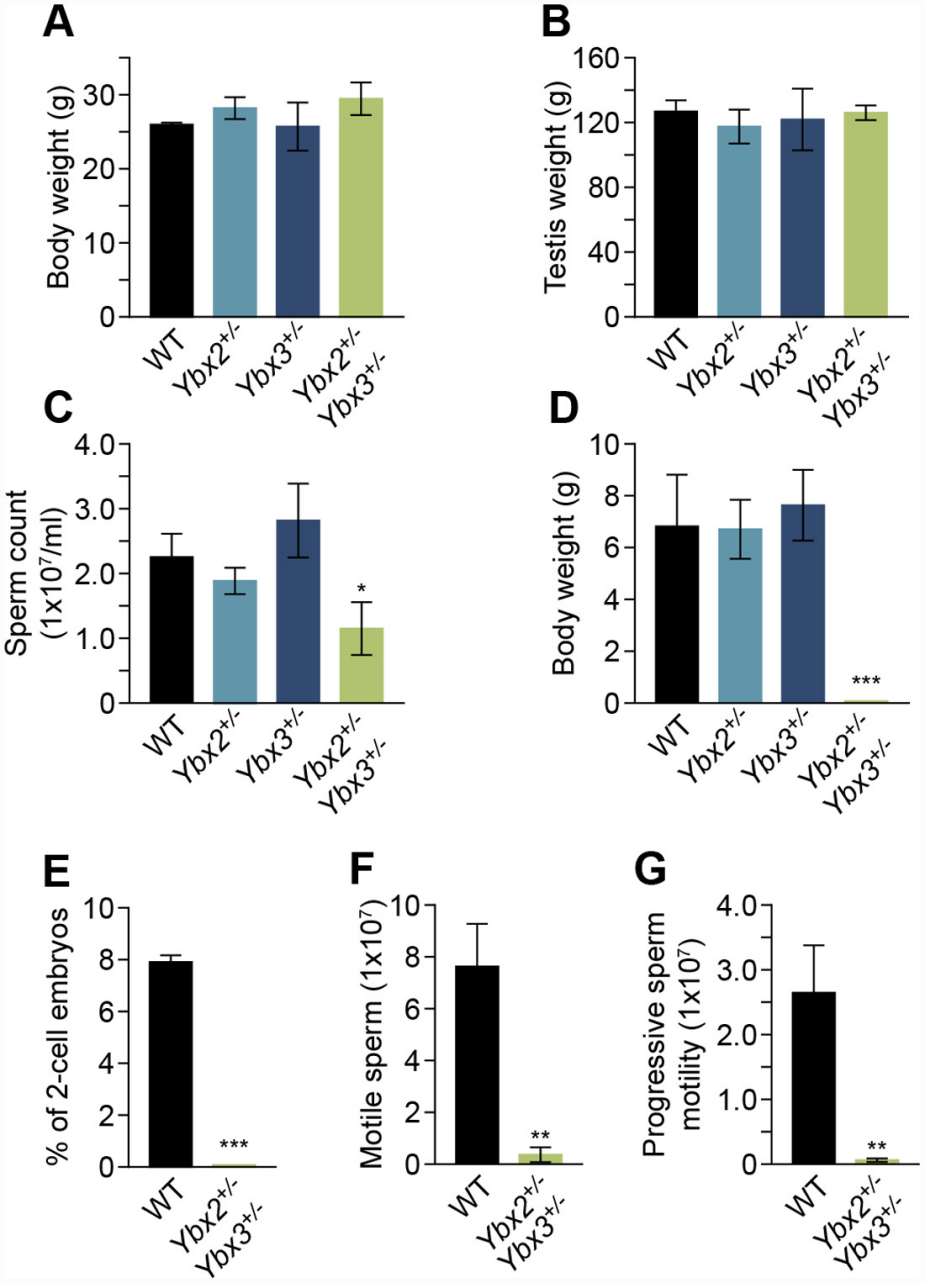
Reproductive parameters of compound heterozygous mutant mice. (A) Body weight and (B) testis weight remained unchanged, while (C) epididymal sperm count is significantly decreased in compound heterozygous relative to single heterozygous (*Ybx2* ^+/-^ or *Ybx3* ^+/-^) or wildtype mice. Values are mean ± SD, N = 3, * indicates p < 0.05. Fertility assessed by (D) embryos produced from mating with C3H/HeJ fertile females and sperm function quantified by (E) motile sperm and (F) progressive sperm motility assessed by computer-assisted sperm analysis demonstrating defects in sperm motility in compound heterozygotes. The data are expressed as percentage of motile sperm ± SD. ** p < 0.01 relative to wildtype sperm. N = 3. (G) 2-cell embryos derived from *in vitro* fertilization (IVF) of oocytes from superovulated C3H/HeJ females demonstrating infertility in compound but not single heterozygous males. The data are expressed as number of embryos ± SD per dam or percentage ± SD of 2-cell embryos. N = 3, *** p < 0.001.

To examine possible redundancy in YBX2 and YBX3 function, we attempted to generate double homozygous null mice starting with reciprocal *Ybx2* ^+/-^ *Ybx3* ^+/-^ crosses. Compound *Ybx2/3* heterozygous males were obtained in the expected Mendelian frequencies. However, intercrosses of compound *Ybx2/3* heterozygotes to generate double homozygotes consistently failed to produce any offspring, suggesting that either double homozygosity was lethal, or that one or both parental genotypes were infertile. To distinguish between these possibilities, we first assessed the fertility of single heterozygous (*Ybx2* ^+/-^ or *Ybx3* ^*+/-*^) and compound *Ybx2/3* heterozygous (*Ybx2* ^+/-^; *Ybx3* ^+/-^) males (Fig 2). We found that compound but not single heterozygous males had significant reductions in sperm numbers relative to controls, despite having normal body and testis weights (Fig 2A–2C). *In vivo* fertility tests demonstrated that while compound *Ybx2/3* heterozygotes were capable of producing plugs, no offspring were produced (Fig 2D). To further explore potential causes of sterility in compound *Ybx2/3* heterozygotes, sperm motility was assessed. This analysis demonstrated significantly reduced levels of total motile and progressively motile sperm in compound *Ybx2/3* heterozygotes relative to controls (Fig 2E and 2F). Additionally, *in vitro* fertilization demonstrated *Ybx2/3* heterozygous sperm were not capable of producing 2-cell embryos (Fig 2G), demonstrating the fertility defect observed in compound *Ybx2/3* heterozygotes is due in part to fertilization defects.

### Compound heterozygotes show normal YBX localization patterns

We next sought to determine if compound heterozygosity of *Ybx2* and *Ybx3* modified the amount or localization pattern of YBX2 and YBX3 in adult testis. By immunofluorescence, we observed no clear difference in either YBX2 or YBX3 localization in adult wild type, single heterozygous, and compound *Ybx2/3* heterozygous testes (S3A and S3B Fig). However, western blot analysis revealed reduced protein levels of both YBX3 isoforms in *Ybx3* heterozygote testes (S3C Fig) and a similar reduction of YBX2 in *Ybx2* heterozygote testes (S3D Fig). Compound *Ybx2/3* heterozygote testis had YBX2 and YBX3 protein levels reduced relative to wild type and similar to those observed in the respective single heterozygotes.

### Sperm from compound *Ybx2/3* heterozygous mice have abnormal morphology, decreased chromatin integrity, and reduced PRM2 processing

**Fig 3:**
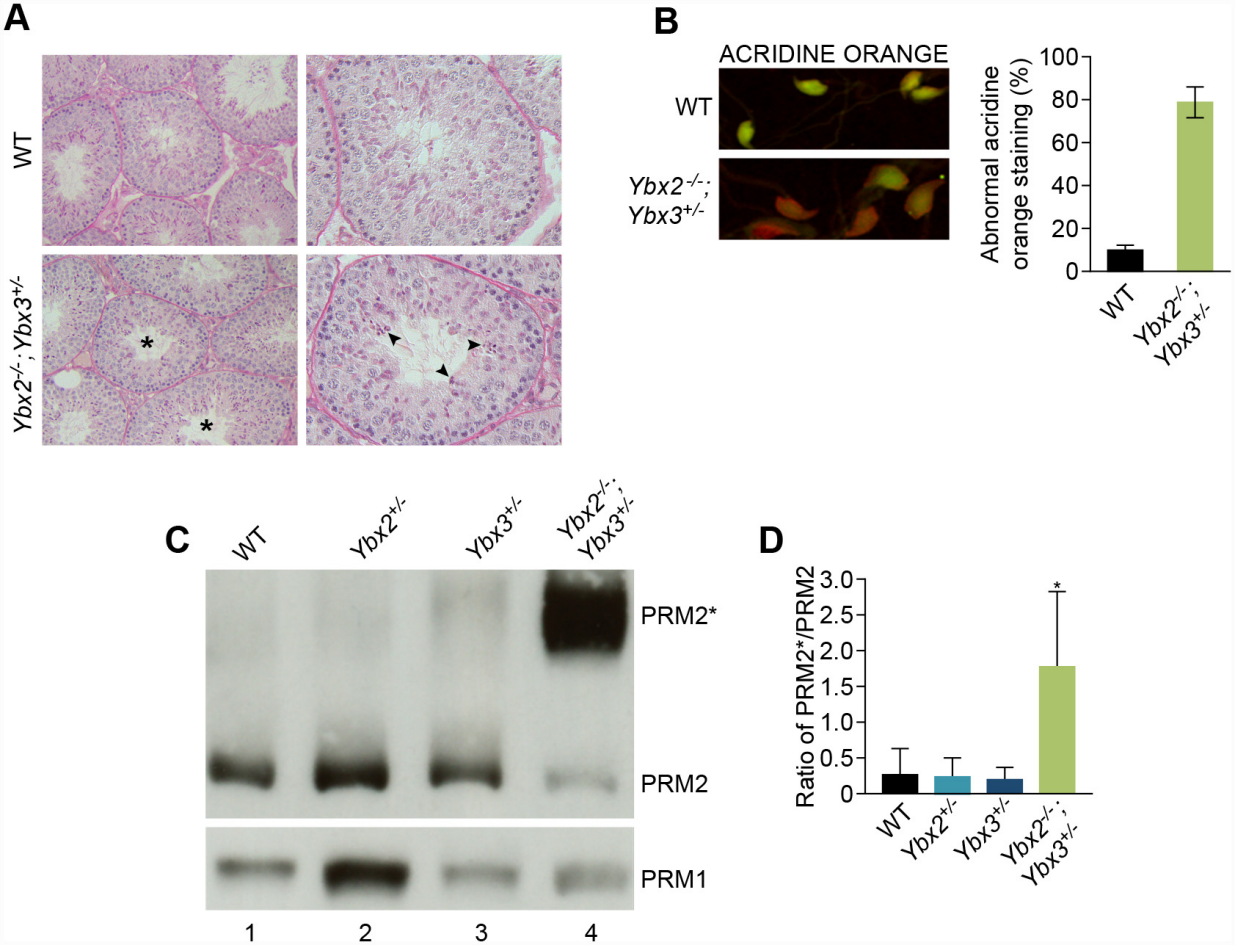
Morphological analysis of spermatogenesis and sperm chromatin condensation in compound heterozygous mutant mice. (A) Periodic acid–Schiff–stained sections of adult testis shows tubule cross-sections containing abnormal elongating spermatids (asterisks). Step 9 spermatids demonstrate defective elongation and abnormal condensation within tubule cross-sections (arrowheads). (B) The presence of single stranded DNA indicative of DNA damage was evaluated by sperm acridine orange staining. In compound heterozygous mutant sperm acridine orange fluoresced in a manner consistent with single-stranded DNA. Quantification of acridine orange–stained sperm demonstrating increases in compound heterozygous relative to wildtype sperm. Values expressed as a percentage ± SD. N = 3. p = 0.052. (C). Western blot analysis confirming the presence of PRM2* in compound heterozygous mutant sperm, while mature PRM2 levels were decreased (Lane 4). A representative blot is shown (N = 3). PRM1 levels remained unchanged (Lower panel). (D) Quantitation of the PRM2*/PRM2 ratio by densitometric scan of the PRM2 western blot revealed a significant increase in PRM2*/PRM2 ratio in compound heterozygous mutant relative to wildtype or *Ybx2* ^+/-^ or *Ybx3* ^+/-^ mutants. p< 0.05, N = 3.

Histological examination of compound *Ybx2/3* heterozygote adult testes revealed defects in spermatid elongation relative to controls (Fig 3A). Additionally, globozoospermia (round-headed sperm) was frequently observed in sperm from compound *Ybx2/3* heterozygotes (S4 Fig) but not controls. Sperm isolated from compound *Ybx2/3* heterozygotes revealed abnormal DAPI staining of heterochromatin (arrowhead, S4A Fig) not observed in control sperm, suggesting that chromatin organization was affected. This was confirmed by staining with acridine orange, the fluorescent signature of which can be used to detect single stranded DNA, an indirect measure of chromatin compaction (Fig 3B). This analysis revealed abnormal chromatin compaction in sperm from compound *Ybx2/3* heterozygotes compared to controls.

PRM1 and PRM2 are key modulators of chromatin compaction, thus they were assessed by napthol blue staining (S3C Fig) and western blotting of total basic proteins from sonication-resistant sperm nuclei (Fig 3) in *Ybx2/3* heterozygotes. Moderate decreases in PRM1 level were observed in compound *Ybx2/3* heterozygote spermatozoa. In contrast, both napthol blue staining and western blotting revealed abnormal PRM2 specifically in compound *Ybx2/3* heterozygote epididymal sperm relative to wild type (Fig 3C).

PRM2 is synthesized as a precursor of 107 amino acids (PRM2*) that undergoes proteolytic cleavage after associating with DNA to yield a mature 63 amino acid peptide [3]. Decreased PRM2* processing has been associated with decreased nuclear maturity [17]. Compared to wild type or single heterozygous epididymal sperm, we observed a decrease in PRM2 concomitant with an increase in PRM2* in compound *Ybx2/3* heterozygous sperm. In addition, densitometric quantitation of western blots revealed a significant increase in the PRM2*: PRM2 ratio in compound heterozygotes (Fig 3D) relative to all other genotypes, indicating decreased nuclear maturation in the compound *Ybx2/3* heterozygote sperm.

### Minimal changes in transcript abundance occur in compound *Ybx2/3* heterozygote testes

In order to determine if the compound *Ybx2/3* heterozygous phenotype could be attributed to changes in gene expression; we performed RNA sequencing (RNA-seq) of total RNA from control, single, and compound heterozygote testes (S5 and S6 Figs). This analysis demonstrated relatively minimal impact on total gene expression in either single or compound heterozygote testes relative to controls. Gene expression changes that were observed in the compound *Ybx2/3* heterozygote were not limited to those observed in one or both single heterozygotes (S7A Fig). Those genes with altered expression in compound *Ybx2/3* heterozygotes were expressed predominantly in the spermatocyte and spermatid cell populations (S7B Fig), in which YBX2 and YBX3 are expressed. In several cases, including *Prm1*, *Prm2*, *Tnp1*, and *Tnp2*, transcript abundance is slightly reduced in compound heterozygotes relative to wildtype, likely a consequence of changes in cellular composition due to loss of post-meiotic cells as opposed to YBX-driven changes in transcription. These observations suggest compound *Ybx2/3* haploinsufficiency has only minimal impacts on testis transcript abundance.

### Testes of compound YBX haploinsufficient mice display altered polysome occupancy

**Fig 4:**
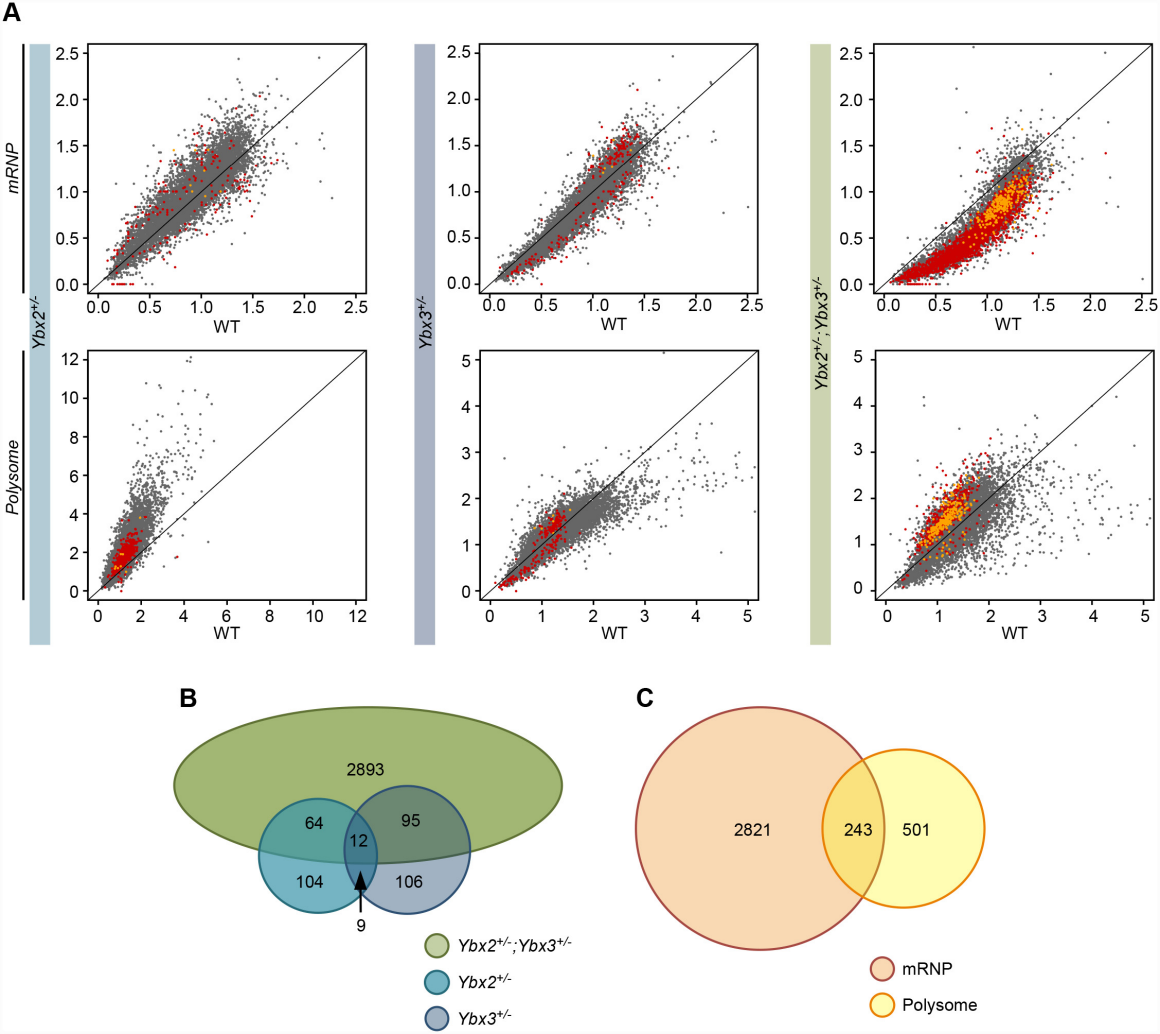
Testis transcript mRNP and polysome occupancy in single and compound heterozygotes. (A) Comparison of mutant versus wildtype mRNP or polysome abundance of expressed transcripts demonstrating global decreases in mRNP abundance and global increases in polysome abundance in compound heterozygotes but not single heterozygotes relative to wildtype. Data represented as mRNP or polysome abundance normalized by total RNA abundance and averaged across biological replicates. Red points—transcripts with significant differences between wildtype and mutant (t-test, N = 2, p-value < 0.05) within either the mRNP or polysome fractions. Yellow points—transcripts with significant differences between wildtype and mutant in both mRNP and polysome fractions. (B) Intersect of transcripts with differential mRNP or polysome occupancy in the three mutant models demonstrating limited overlap between the single and compound heterozygotes. (C) Intersect of transcripts with differential mRNP and polysome occupancy in compound heterozygotes. Transcripts within the overlap represent high confidence targets for abnormal translation repression.

Both YBX2 and YBX3 have been proposed to act as translational repressors by sequestering target transcripts in translationally inactive mRNP particles of post-meiotic germ cells [6]. Thus, the impact of compound *Ybx2/3* haploinsufficiency on transcript mRNP and polysome occupancy in the testis was assessed globally in control, single, and compound heterozygote testes by RNA sequencing (S5 and S6 Figs). After normalization, mRNP and polysome occupancy was calculated as the ratio of mRNP or polysome signal to total signal for each expressed gene. Comparison with wildtype controls (Fig 4A) demonstrated that *Ybx2* single heterozygotes had a slight increase in polysome occupancy, while compound *Ybx2/3* heterozygote testes had substantially reduced mRNP occupancy concurrent with increased polysome occupancy in a subset of genes. Comparison of impacted genes between single and compound *Ybx2/3* heterozygote testes demonstrated the majority of genes with altered mRNP or polysome occupancy in compound heterozygotes were not impacted in either single heterozygote (Fig 4B). 243 genes were significantly altered in both the mRNP and polysome fractions of the compound *Ybx2/3* heterozygotes (Fig 4C). Of these, only two were similarly altered in either of the single heterozygotes.

### Compound but not single heterozygote testes demonstrate widespread loss of translational repression

**Fig 5:**
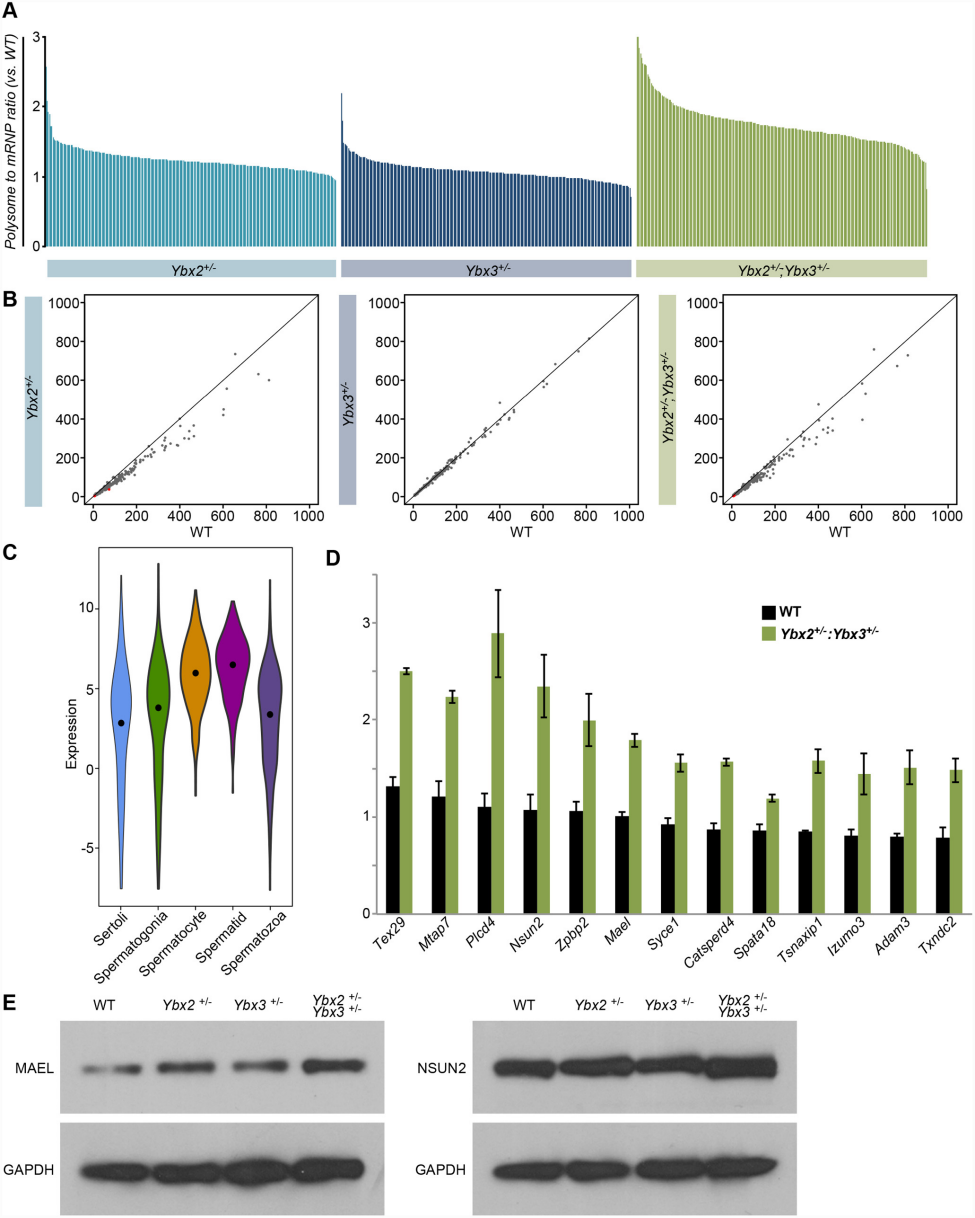
Translational repression and expression of high confidence targets. (A) Ratio of polysome to mRNP occupancy across genotypes as compared to wildtype demonstrating substantially higher polysome occupancy in compound heterozygotes when compared to single heterozygotes. Data represented as average across biological duplicates. (B) Expression of transcripts with altered mRNP and polysome occupancy compared across genotypes demonstrating minimal change in overall expression demonstrating altered polysome occupancy is not driven by differences in total expression. (C) Cell-type expression of high confidence targets demonstrating enriched expression in YBX2 and 3 positive cell types. Expression—log_2_(average TPM). Black dot—median value. (D) Polysome to mRNP ratio of transcripts relevant to spermatogenesis showing increased polysome occupancy in compound heterozygotes relative to wildtype. Note the only transcript with a well-characterized YRS is *Spata18*. Data is represented as the average polysome to mRNP ratio ± SD. (E) Western blot detection of MAEL, NSUN2, and GAPDH in whole adult testis protein lysates from wildtype, single heterozygote, and compound heterozygous mutants demonstrating increase MAEL and NSUN2, but not GAPDH, abundance in the compound heterozygous mutant testis relative to wildtype.

Given the proposed role of the YBX proteins as translational repressors, we aimed to demonstrate the directionality of change for those genes with abnormal polysome and mRNP occupancy in compound *Ybx2/3* heterozygote testes. Polysome-associated RNAs represent actively translated transcripts while mRNP associated RNAs represent translationally inactive transcripts; thus, the polysome to mRNP ratio is an approximate measure of translational activity, with higher ratios indicating higher target translation. Comparison of the wildtype and compound *Ybx2/3* heterozygote polysome to mRNP ratio for each impacted gene demonstrated the ratio was invariably greater in compound *Ybx2/3* heterozygote testes relative to control (Fig 5A), a pattern that did not hold true for either single heterozygote. The overall transcript abundance of genes with abnormal polysome and mRNP occupancy in compound *Ybx2/3* heterozygote testes was not changed in either single or compound heterozygote testes (Fig 5B). However, impacted genes are predominantly expressed in cell types that also express YBX2 and YBX3, spermatocytes and spermatids (Fig 5C). A number of these genes (Fig 5D) play important roles in meiotic and post-meiotic germ cell development and thus may be giving rise to the specific phenotypic defects observed in compound *Ybx2/3* heterozygotes.

To validate and support these findings, two genes with altered polysome occupancy in the compound heterozygotes were examined at the protein level (Fig 5E). Both the homolog of Drosophila maelstrom (MAEL) and NOL1/NOP2/Sun domain family member 2 (NSUN2) are detectible in pachytene spermatocytes and mutation of either results in meiotic and post-meiotic germ cell differentiation defects [18,19]. In both cases, total mRNA abundance assessed by RNA-seq showed no variation with genotype. However, polysome occupancy was significantly increased in the compound heterozygous mutant testis relative to wildtype. Western blot detection and densitometric quantification showed MAEL protein to be 1.46 times and NSUN2 protein to be 1.76 times more abundant in the compound heterozygous mutant testis relative to wildtype, indicative of decreased translational repression. Based on these observations we conclude the abnormal mRNP and polysome occupancy observed in compound *Ybx2/3* heterozygotes is due to a cell-type specific loss of translational repression.

### Loss of translational repression in compound *Ybx2/3* heterozygote testes is inversely correlated to the presence of a YRS

**Fig 6:**
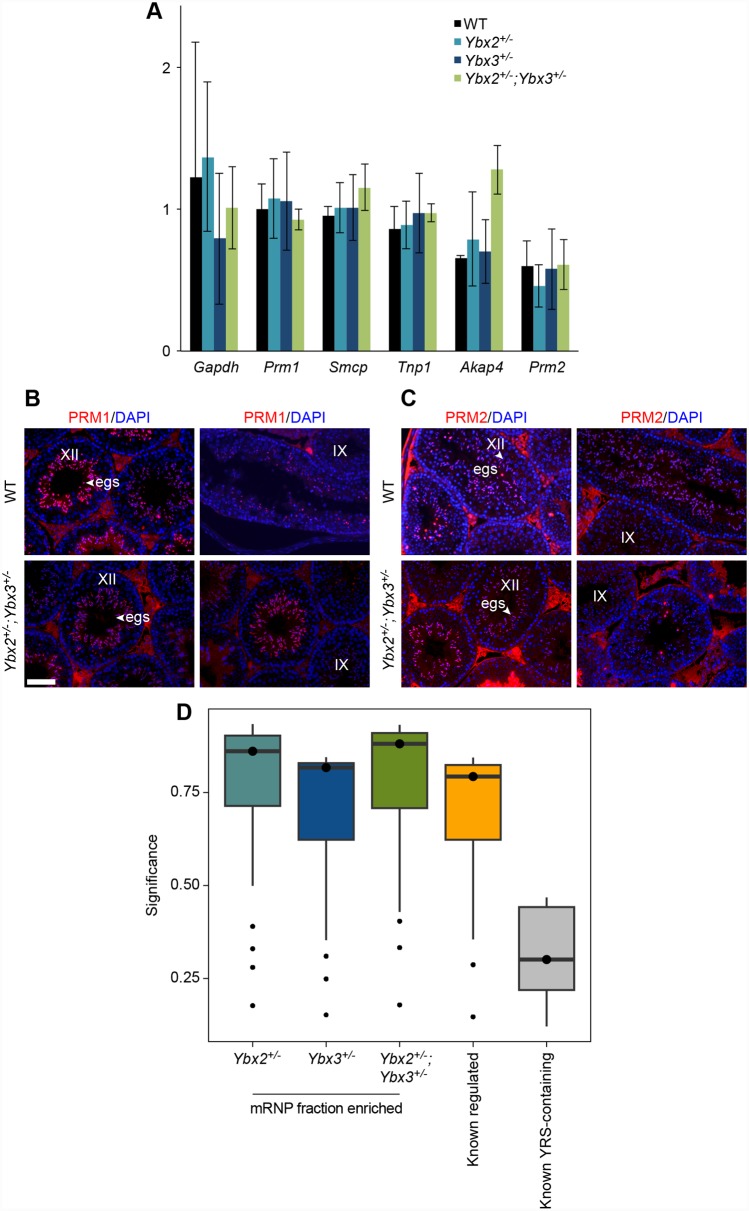
YRS-independent regulation in compound heterozygote testes. (A) RNA-sequencing derived polysome to mRNP ratio of known YRS-containing transcripts across wildtype and heterozygote testes demonstrating no impact on polysome occupancy in compound heterozygotes. Data is represented as the average polysome to mRNP ratio ± SD. (B) Stage specific expression of PRM1 and (C) PRM2 in elongating spermatids (arrowhead) in wildtype and compound heterozygous testis. *Prm1* and *Prm2* mRNAs are not prematurely translated in compound heterozygous mutant mice as shown by the absence of PRM1 staining in stage IX (step 9 elongating spermatids). Scale bar = 100 μm. egs: elongating spermatid. (D) Significance of degenerate YRS motifs identified in the 3’ UTR of transcripts with altered mRNP abundance across genotypes. Comparison with known YRS-containing UTRs demonstrates substantial under-representation of high confidence YRSs in mis-regulated transcripts.

YBX proteins are thought to regulate translation via YRS elements within repressed messages [12,13]. Many transcripts required for post-meiotic germ cell differentiation events, like chromatin compaction and flagellar development, contain YRS elements and are known to be under translational regulation [12]. However, only one transcript (*Spata18*), commonly considered to be YRS-regulated, displayed altered mRNP or polysome occupancy in compound *Ybx2/3* heterozygote testes (Figs 5D and 6A). Immunofluorescent detection of PRM1 and PRM2, the protein products expressed from two well-established YRS containing transcripts did not show premature translation. In control testes, PRM1 and PRM2 are first observed in step 10 spermatids (stage X) with maximal staining intensity in step 12 spermatids (stage XII). A similar pattern was observed in compound heterozygous testes (Fig 6B and 6C), demonstrating no aberrant translational activation. In contrast, both MAEL and NSUN2 appear to have decreased translational repression in compound heterozygous testes (Fig 5E) and neither mRNA is enriched for YRSs based on guided motif analysis.

Given this finding, we asked whether those genes with aberrant mRNP and polysome occupancy in compound *Ybx2/3* heterozygote testes contained canonical YRSs, previously defined as [TAC][CA]CA[TC]C[ACT] [13]. To do so, we applied the same directed motif analysis to a set of UTRs known to contain a canonical YRS (YRS-containing) [12] and UTRs derived from genes misregulated in the mRNP fraction of either single or compound *Ybx2/3* heterozygous testes (compound heterozygous regulated). For each putative YRS, a significance value was calculated and these values compared across all UTR sets (Fig 6D). A q-value threshold of 0.2 identified at least one YRS in 90% of the YRS-containing transcripts whereas this same threshold identified high confidence YRSs in only 11.9, 14.0, and 16.4% of genes with altered mRNP occupancy in *Ybx2*, *Ybx3*, and compound *Ybx2/3* heterozygote testes, respectively. However, for those transcripts containing a canonical YRS, the average number of YRSs per transcript was similar across all datasets, with a range of 2.4 to 2.89 canonical YRSs per regulated transcript. These results demonstrate only a small fraction of single or compound heterozygous regulated transcripts contain high confidence YRSs, suggesting preferential binding of Y box proteins to YRS-containing mRNAs in compound *Ybx2/3* heterozygote testes.

## Discussion

Temporal translational control is fundamental to post-meiotic germ cell-differentiation and Y-box proteins are known regulators of this process. Two such proteins, YBX2 and YBX3, are highly expressed in post-meiotic male germ cells and have been theorized to have similar functions as negative regulators of translation. However, there have been mixed reports regarding their exact molecular function and it has been unclear whether they 1) repress translation and/or prevent degradation of repressed mRNAs, 2) function independently or together, and 3) bind RNA specifically and/or non-specifically. Comparison of single and compound heterozygotic models allowed us to discern between YBX2 and YBX3 targets in a novel context, yielding new insights into their function and the rules governing YBX target selection.

Previous work assessing the translation profiles of putative Y-box protein targets in germ cells suggested that both YBX2 and YBX3 act by repressing translation [4,15]. Consistent with this hypothesis, we observed substantial loss of translational repression in compound *Ybx* heterozygote testes. Although several reports have implicated Y-box proteins in conferring message stability [16,20], particularly for those messages that undergo translational repression [16], we failed to observe a reduction in mRNP occupancy without a commensurate increase in polysome occupancy in any genotype, and genes with impacted polysome occupancy in the compound *Ybx2/3* heterozygote testes had little or no change in overall abundance. As those models reported to have substantial gene abundance alterations also display cellular composition changes [21] not present in our model, we conclude that the primary role of Y-box proteins in a nearly intact cellular context is translational repression and not message stability.

Although a large number of transcripts with an altered mRNP/polysome distribution were observed in *Ybx2/3* heterozygote testes, we observed only minor loss of translational repression on a small number of targets in single *Ybx* heterozygote testes. Likewise, fertility in male *Ybx2/3* heterozygotes was severely impacted while no such impact was observed in single *Ybx* heterozygotes. Given this, we propose that the loss of translational repression in the compound *Ybx2/3* heterozygote testes is the main driver for the observed infertility phenotype.

The vast majority of genes with abnormal polysome and mRNP occupancy in the compound *Ybx2/3* heterozygote testes do not show abnormalities in the single *Ybx2* or *Ybx3* heterozygote testes. Previous reports have shown RNA-dependent YBX2 and YBX3 interaction within the mRNP [6], suggesting they may function together to attain target repression. This notion is supported by non-additive impacts on gene expression and translational repression observed in compound *Ybx2/3* heterozygote testes. We conclude these targets depend upon the overall amount of YBX2/3 and propose that YBX2/3 act together to repress translation of the same target mRNAs.

The sequence dependence for YBX target selection has been an ongoing question in the field. Early reports suggested the mechanism of YBX target selection is sequence independent [5,22,23], while more recent work has demonstrated YRS-dependence for YBX-mediated translational repression [13]. *In vitro*, this dependence appears to be directly correlated to protein concentration. YBX proteins at high concentrations bind non-YRS and YRS-containing RNAs similarly, however as YBX protein concentration drops binding capacity of non-YRS RNAs is lost much more rapidly than for YRS containing RNAs [24]. Additionally, YRS-containing RNAs more successfully compete for YBX protein binding than non-YRS RNAs [13].

*In vivo*, the shift in mRNP/polysome distribution of non-YRS containing RNAs in *Ybx2/3* compound heterozygotes suggests that YBX proteins can also bind RNA independent of YRS elements. At first glance, the seemingly paradoxical finding of near complete resistance to changes in mRNP/polysome distribution of YRS-containing mRNAs under reduced protein conditions would seem to suggest that YRS elements do not influence YBX translational repression. However, these findings lead us to propose a model whereby at high YBX-to-target-RNA ratios Y-box proteins are capable of maintaining repression of both YRS containing and non-YRS targets. Under limited Y-box protein conditions, YRS-containing targets are preferentially sequestered, leading to translation of mRNAs lacking YRSs. This model is bolstered by other *in vivo* observations indicating sequence-independent functions of Y-box proteins, particularly in the context of high protein to target ratio as occurs in normal spermatogenic cells [20]. This protein to target ratio is reduced in the haploinsufficient state. We argue that intact repression of previously defined YRS-containing mRNAs [12] in *Ybx2/3* compound heterozygotes is exactly what one would predict from previous *in vitro* studies [24], providing strong support to the notion that YBX2/3 bind RNA hierarchically, with YRS-containing mRNAs as the favored targets.

Nonetheless, it is likely that hierarchical RNA binding by YBX proteins is modulated by additional factors beyond the YRS. Previous reports have demonstrated that deletion of the YRS in the 3’UTR of *Prm1* does not lead to translational activation and that the ability of a YRS to confer translational repression is context dependent [25], suggesting other RNA elements, YRS context, or other factors bound to other regions of the RNA, can influence YBX behavior. The models and data generated in this work will provide useful tools to unravel the exact elements that regulate YBX-mediated translational repression.

It has long been recognized that mutation of single genes sometimes fails to result in a phenotype, yet in combination with a second-site mutation is lethal. This synthetic lethality can result from both gene redundancy as well as from non-redundant additive or synergistic effects [26]. Compound, but not single, heterozygosity of *Ybx2* and *Ybx3* results in male sterility, demonstrating a similar mechanism is at play between two Y-box proteins in the male germ cell. Given both YBX1 and YBX3 expression is widespread during development [10], this new understanding of Y-box protein behavior may provide insight into their function in other systems. Additionally, the “synthetic sterility” observed in compound *Ybx2/3* heterozygotes suggests that for some of the roughly one in ten couples facing male-derived infertility, heterozygosity at two or more loci may be a driving factor.

## Materials and Methods

### Animals

Protocols using mice in this study were approved by the Jackson Laboratory Animal Care and Use Committee (Permit Number: 07007) and are in accordance with the “Guide for the Care and Use of Experimental Animals” established by National Institutes of Health (NIH) (1996, revised 2011). Animals were maintained in a 12 h light and 12 h dark cycle vivarium in the Research Animal Facility at The Jackson Laboratory. Autoclaved NIH31 diet (6% fat) and HCl acidified water (pH 2.8–3.2) were provided *ad libitum*.

### Generation of *Ybx3* ^*tm1Reb*^ and production of *Ybx3-* carrying mice

The 8 kb *Msy4* genomic locus was replaced by a targeting construct containing a 5’LoxP site inserted between exons 1 and 2 and a 3’LoxP site inserted between exons 3 and 4 of mouse *Ybx3*. Amplified DNA was sub cloned into the pK-11 vector with neomycin (G418) as a selectable marker. The resulting targeting vector was linearized using Not I and electroporated into ES (A3) cells. G418 resistant clones were selected for homologous recombination and were verified for proper targeting by Southern blot analysis. We generated chimeric mice by injecting embryonic stem cell clones into blastocysts followed by embryo transfer in 129S4/svJaeJ female. Chimeric male offspring were bred with 129S4/svJaeJ females to generate F1 heterozygous (*Ybx3* ^+/-^) mice. The heterozygous females were intercrossed to heterozygous males to generate homozygous (*Ybx3* ^tm1Reb^) null mice.

### Generation of *Ybx2* ^+/-^; *Ybx3* ^+/-^ mice

Single heterozygous (*Ybx2* ^*tm2Nbh*^) mice in 129S5 / SvEvBrd hybrid genetic background was kindly provided by Dr. M. M. Matzuk (Baylor College of Medicine). Compound heterozygous mice used for this study were generated by breeding *Ybx3* ^+/-^ females with *Ybx2* ^+/-^ male mice although reciprocal crosses resulted in the same compound heterozygous phenotype. Genotyping was accomplished by PCR amplification of *Ybx2* and *Ybx3* using specific primers (S1 Table).

### Southern blot analysis

Genomic DNA was digested with Hpa I and resolved on a 0.8% TAE Agarose gel at 100V for 5 h. Southern blotting was performed using a standard protocol. DNA was UV cross-linked to a Hybond nylon membrane (Amersham). The membrane was incubated in prehybridization buffer (6X SSPE (150 mM sodium chloride, 10 mM sodium phosphate and 1 mM EDTA), 1% Sarkosyl and 0.1% Bovine serum albumin, BSA) at 65°C for 1h. Following prehybridization, the membrane was incubated at 65°C overnight in hybridization buffer (6X SSPE, 1% Sarkosyl, IX Denhardt’s reagent containing 100 μg/ml salmon sperm DNA) and denatured probe (pGEM-Ex4.5 EcoRI-digested 500 bp DNA) that was labeled using α-^32^P dCTP (GE Healthcare Biosciences) and a random primer-labeling kit (Promega). The membrane was washed 2X in Buffer 1 (2X Saline-sodium citrate buffer, SSC, 1% SDS) at 65°C for 20 minutes and 2X in Buffer 2 (0.1 X SSC, 0.1% SDS) at 42°C for 20 minutes. The membrane was exposed to X-ray film (X-OMAT Blue XB, PerkinElmer Life Sciences) for a week and developed.

### Northern blot analysis

Total RNA was extracted from the adult testis using Trizol reagent (Life technologies) followed by purification of total RNA using the PARIS kit as per manufacturer’s instructions (Ambion). Ten micrograms of total RNA was resolved on a 1% formaldehyde agarose gel and transferred overnight to a Hybond nylon membrane using 20X SSC. The membrane was then UV-crosslinked and incubated in prehybridization buffer (Clontech) containing 50% formamide, 6X SSPE, 5X Denhardt’s buffer, 1% SDS and 100 μg/ml of denatured salmon sperm DNA) for 2 h at 68°C. The membrane was incubated in hybridization buffer containing denatured probe (pGEM-3Z *Msy4)*, labeled using α-^32^P dCTP and a random primer labeling kit, at 68°C overnight. The blot was washed 3x in 2X SSC containing 0.05% SDS for 15 min and twice in 0.1X SSC containing 0.1% SDS) at 50°C for 20 minutes. The membrane was exposed to X-OMAT Blue XB film for 1 h and developed. The membrane was then stripped in boiling 0.5% SDS solution, cooled, rinsed in 2X SSC and probed using α-^32^P dCTP-labeled β-Actin probe (pUC-β actin) and developed by autoradiography.

### Immunoblotting analysis of YBX2, YBX3, MAEL, and NSUN2 proteins

Adult testis was pulverized into fine powder on dry ice using mortar and pestle. For YBX detection, powders were homogenized in Dignam buffer (10 mM HEPES pH 7.6, 1.5 mM MgCl_2_, 10 mM KCl) containing protease inhibitors, including complete protease inhibitor cocktail tablet (Roche) and additional protease inhibitors including 1 μg/ml of pepstatin A, 0.2 μM phenylmethylsulfonyl fluoride (PMSF) and 0.5 mM dithiothreitol (SIGMA Aldrich). Protein extracts prepared in Laemmli buffer were boiled and electrophoresed in 8% SDS-polyacrylamide gels. For MAEL and NSUN2 detection, powders were dissolved in SDS loading buffer minus dithiothreitol (100 mM Tris-Cl, pH 6.8; 4% SDS, 0.2% bromophenol blue, 20% glycerol) and boiled after the addition of 200 mM dithiothreitol followed by electrophoresis on a 10% SDS-polyacrylamide gel. In all cases, the proteins were transferred to an Immobilon PVDF membrane (Millipore). After transfer, the membrane was blocked for an hour in 5% non-fat dry milk in Tris- buffered saline (TBS) and then incubated in primary antibodies overnight at 4°C. Primary antibodies used here include rabbit polyclonal anti-YBX3 [6] (1:5000), rabbit polyclonal anti-YBX2 (a C-terminal-specific antibody raised against the peptide sequence (GPTDGSRPEPQRPRN) (Life Technologies), rabbit anti-NSUN2 (ProteinTech), rabbit anti-MAEL [18] (a gift from Dr. Alex Bortvin), rabbit anti-GAPDH (Cell Signaling Technology) and horseradish peroxidase (HRP)–conjugated mouse anti-β-Actin (SIGMA) (1:25,000). The membrane was washed three times in TBS with 0.05% Tween-20 (TBST) and incubated in secondary antibody, goat anti–rabbit IgG-HRP (Bio-Rad Laboratories)(1:5000) in TBS buffer at room temperature for 2 h. Following 3x washes in TBS buffer, the membrane was developed using an ECL western blotting detection kit (GE Healthcare Biosciences, RPN2109). Densitometric analysis was completed using the gel plot options of ImageJ (http://imagej.nih.gov/ij/) and was assessed for multiple exposures per protein to ensure accuracy.

### Histological and immunofluorescence analysis

Testes were dissected from adult mice and incubated overnight in Bouin’s fixative before embedding in paraffin wax. Sections (5 μm) were stained with Periodic Acid–Schiff’s (PAS) reagent. For immunofluorescence studies, sections were deparaffinized in xylene and rehydrated without antigen retrieval. Tissue sections were blocked in PBS containing 3% normal goat serum and then incubated with primary antibodies overnight at 4°C. Primary antibodies were used at the following dilutions: YBX3 (1:5000) and YBX2 (1:2000). Following 3x washes for 5 minutes in PBS, the sections were incubated at room temperature for an hour with goat anti-rabbit IgG or goat anti-mouse conjugated to Alexa Fluor R568 (Life Technologies)(1:1000) in PBS containing 3% normal goat serum. Following 3x washes in PBS in the dark, sections were mounted with Vectashield medium containing DAPI (Vector Laboratories). Fluorescence was imaged using Zeiss Axioscop microscope with filterset 10 and formatted using Photoshop software (Adobe Systems).

### Fertility testing

Adult compound heterozygous mutant and wild type littermate control male mice were mated with fertile C3H/HeJ females (6–8 weeks old) and scored for vaginal plugs. At embryonic (E) day 12.5–14.5, the females were euthanized and the embryos per dam were determined for each sire mating.

### Sperm count and morphology

Epididymides were dissected from adult mice and diced in 1 ml of phosphate buffered saline (PBS). The diced tissue was incubated at 37°C for an hour to release sperm that were then diluted 1:10 in PBS and counted using a hemocytometer. Duplicate counts were evaluated for each mouse sample (N = 3–6) and expressed as mean ± S.D. Sperm morphology was assessed by mounting spematids in VectaShield with DAPI (Vector Laboratories; Burlingame, CA), and imaging the spermatids on a Leica SP5 laser scanning confocal microscope with a 63x/1.3NA glycerol objective. The image series were deconvolved and rendered using the Amira 4.1.2 software package (Visage Imaging; San Diego, CA). A heat‐map colorimetric was used, where DAPI intensity is represented on a red to bright yellow heat map.

### Sperm motility

Sperm motility was determined using a Computer-Assisted Semen Analyzer (CASA) (IOVS II, Hamilton Thorne). Epididymal sperm were incubated in fresh MEM medium (Life Technologies) containing Earle’s balanced salt solution supplemented with both essential and nonessential amino acids, 0.23 mM pyruvic acid, 75 mg/L penicillin G, 50 mg/ml streptomycin sulfate (Life Technologies), and 0.01 mM tetra-sodium EDTA, 3 mg/ml BSA and 5% fetal bovine serum (Sigma-Aldrich). The motility of at least 100 spermatoazoa was determined and progressive sperm motility, the ability of the sperm to swim fast in a straight line, was recorded.

### *In vitro* fertilization

Epididymal sperm from compound double heterozygous males and wildtype littermate controls was used to fertilize superovulated wildtype (C3H/ HeJ) oocytes. Fertilization was carried out for 4 h in the same medium as used for assaying sperm motility (described above). The procedure was carried out at 37°C in a modular incubation chamber (Billups Rothenberg) in a gas-infused atmosphere composed of 5% O_2_, 5% CO_2_ and 90% N_2_. The pronuclear embryos were washed twice in fresh medium and cultured overnight in 1 ml of fresh medium. Embryos were scored at 48 hours and results were expressed as a percentage of 2-cell embryos.

### Isolation of basic nuclear proteins

Basic nuclear proteins as well as PRM1 and PRM2 from the cauda epididymis and testis were isolated as described [27]. Total protein was precipitated from tissue fractions using 20% TCA at -70°C overnight. Samples were centrifuged for 10 minutes at 12,000 rpm and washed twice in 500 μl of 1.0% β-mercaptoethanol (SIGMA Aldrich) in acetone. Protein pellets were air-dried for 20 minutes at room temperature. Basic proteins were dissolved in 20 μl of 5.5 M Urea, 20% β-mercaptoethanol, 0.9% acetic acid (w/v) and 1% Methyl Green (SIGMA Aldrich) for 2h at room temperature. The samples were boiled for 2 minutes and separated on an acetic acid–urea mini gel [28]. Gels were stained to detect basic proteins [29].

### Immunoblotting of basic proteins

Basic nucleoproteins from testes or caudal epididymides were separated by 15% acid urea PAGE and then transferred to a PVDF membrane in 0.7% acetic acid and 1M Urea (Sigma-Aldrich) at 20 volts for 1 h. The membrane was blocked in 5% non-fat milk in 1X PBS (pH 9.0) at room temperature and incubated in Hup1b (PRM1) antibody (1:1000) or Hup 2b (PRM2) antibody (1: 5000) at 4°C overnight (Hup1b and Hup 2b antibodies were generously provided by Dr. Rod Balhorn, Lawrence Livermore National Laboratories). The membrane was rinsed 3x in TBST for 20 minutes and incubated in goat anti-mouse IgG-HRP (1:1000) in 5% non-fat milk in TBST for 2 h at room temperature. Following 3x 10 minute washes in TBST and the membrane was developed using the ECL western blotting detection kit.

### Acridine orange staining of sperm

Sperm (5 x 10^5^ in 50 μl) from the cauda epididymis of adult mice was smeared on a glass slide, fixed in acetic alcohol for 24 h and stained with 0.02% acridine orange prepared in citrate-phosphate buffer, pH 2.5 (SIGMA Aldrich) for 5 min, as described [30]. Following staining, the smears were washed in distilled water and counterstained in Vectashield mounting medium (Vector Laboratories Inc). Imaging was performed using the Zeiss Axioscop microscope with filterset 10 and the following filter combinations: 450–490 nm excitation, 510 nm reflector and 520 nm barrier filters. We examined 100–400 nuclei and scored the fluorescence as green, red or yellow. Acridine orange staining was quantified and expressed as a percentage of total sperm counted.

### Isolation of total, polysome, and mRNP RNAs

Testes from *Ybx2+/-*, *Ybx3+/-*, *Ybx2+/-*:*Ybx3+/-*, and wildtype litermates were isolated at 8 weeks of age in duplicate. Immediately upon collection, testes were immediately placed into lysate buffer and homogenized. Prior to gradient separation, an aliquot of lysate was retained for isolation of total RNA. Sucrose gradient analysis was performed as in [4] with the following alterations. Briefly, lysates were applied to a 10–45% linear sucrose gradient, centrifuged at 36,000 rpm for 2.5 hours in a Beckman Coulter Avanti J-E, and fractions collected on an Isco Density Gradient Fractionator (Model 185).

### RNA sequencing and preliminary processing

Isolated total, mRNP, and polysome RNA was purified via a RNeasy Mini Column (Qiagen) purification. After purification, 2 μl ERCC Spike in Mix 1 (Life Technologies) was added to approximately 100 ng of RNA from each sample. From these mixes, sequencing libraries were constructed using the Stranded Total RNA LT with Ribo-Zero TM Gold Library Prep kit (Illumina) and paired-end 100 bp reads sequenced on an Illumina HiSeq 2500 to a minimum depth of 30 million reads per sample. Reads were aligned to a testis transcriptome via RSEM [31].

### Data normalization and analysis

Expected counts and TPMs were extracted from RSEM and normalized via RUVSeq [32] using the ERCC RNA Spike In Mix (Life Technology) as control input. After normalization, genotype-dependent differentially expressed genes were identified by EBSeq [33] analysis of total RNA samples. Those with a PPDE > 0.95 and TPM > 5 in both replicates of at least one genotype were retained for further analysis. Expression pattern as determined by EBSeq was used to further parse differentially expressed genes. All mRNP and polysome calculations were done on the gene level after filtering for expression (as above), utilized TPMs, and were normalization by total expression within individuals prior to averaging across genotype. Significant differences in occupancy were determined on the gene level by t-test (p < 0.05). Cell-specific expression analysis utilized publically available data (GEO accession numbers GSE43717, GSE43719, and GSE43721 [34]). Individual samples were aligned to a testis transcriptome and expression estimated via RSEM. Motif analysis utilized a guided motif identification tool (FIMO, [35]) that had been provided with the YRS motif identified in [13]. The RNA-sequencing data generated in this work has been deposited in Gene Expression Omnibus (GEO) at http://www.ncbi.nlm.nih.gov/geo/ under accession number GSE73653.

## Supporting Information

S1 Fig Strategy for targeted disruption of the *Ybx3* gene. (A) Schematic representation of the *Ybx3* gene-targeting construct and the targeted *Ybx3* locus is shown. (B) Southern blot analysis the targeted ES ^+/-^ cells revealed homologous recombination of *Ybx3* gene. The wildtype allele (6.7 kb) and the *Ybx3*-targeted allele (8.7 kb) were observed in the ES cells. An unexpected recombination event resulted in insertion of a single loxP site and the neomycin selection cassette upstream of exon 4, leading to disruption of the endogenous *Ybx3* locus. (C) Genotyping using gene-specific primers detected a 900bp and a 450bp product in the heterozygous mutant, a 900bp product in the *Ybx3* ^-/-^ mutant and a 450bp product in wild type mice. (D) Northern blot analysis demonstrates a significant decrease in *Ybx3* transcript (1.8 kb) in *Ybx3* ^-/-^ mutant testis compared to wild type or heterozygous testis. *Actb* was used as a loading control.
(TIF)

S2 Fig Analysis of YBX2 and YBX3 protein in *Ybx2* ^-/-^ mutant and *Ybx3* ^-/-^ mutant testis. (A) YBX2 protein was expressed in round spermatids (rs, arrowhead) at stage VI in *Ybx3* ^-/-^ mutant adult testis. (B) YBX3 protein was detected in the pachytene spermatocytes (ps, arrow) and round spermatids (rs, arrowhead) in *Ybx2* ^-/-^ mutant and wildtype testis. (C) Immunofluorescence staining of adult testis sections with an N-terminal YBX3 antibody reveals a complete absence of YBX3 protein in *Ybx3* ^-/-^ mutant testis. Cytoplasmic staining of YBX3 in pachytene spermatocytes (ps) of wildtype mice is shown (arrow). YBX3 staining in the cytoplasm of the round spermatids (rs) is shown (arrowhead). (D) Complete absence of YBX2 protein in *Ybx2* ^-/-^ mutant testis relative to the wildtype testis. Scale bar = 100 μm.
(TIF)

S3 Fig Analysis of YBX2 and YBX3 protein in compound heterozygous mutant. (A) YBX3 is expressed in late stage pachytene spermatocytes (ps) (arrow) and early to mid-stage round spermatids (rs) (arrowhead) in compound heterozygous mutant testis. (B) YBX2 is expressed in mid-stage pachytene spermatocytes (ps) (arrow) and early to mid-stage round spermatids (rs) (arrowhead) in compound heterozygous mutant testis. (C) Western blot analysis of YBX3 or YBX2 (D) proteins demonstrates a decrease in expression of these proteins in compound heterozygous mutant testis. Scale bar = 100 μm.
(TIF)

S4 Fig Sperm defects in compound heterozygous mutants. (A) Differential interference contrast (DIC) and isosurface rendering of DAPI staining revealed abnormalities in sperm head and tail morphology in compound heterozygous mutant mice (left-hand images). Serial images of individual sperm heads were further resolved using a deconvolution algorithm and then rendered using a red-to-yellow heat map and a fixed color isosurface (right-hand images). The 3D reconstruction of the sperm head (isosurface) revealed abnormal chromatin organization in compound heterozygous mutant mice. (B) Abnormal sperm head morphology was quantified and data expressed as a percentage ± SD. There was a significant increase in the abnormal sperm head morphology in compound heterozygous mutant relative to wildtype mice. N = 3. p < 0.001. (C) Nucleoproteins extracted from 1 x 10^6^ sonication-resistant sperm nuclei was fractionated on 15% Acid Urea PAGE, stained with Napthol blue, imaged using a G-Box Chemi-XT4 Synoptics camera and analyzed by GeneSys software. The positions of protamine 1 (PRM1), mature protamine 2 (PRM2) and the unprocessed PRM2 precursor (PRM2*) are listed. PRM2* was detected only in compound heterozygous mutants (Lane 4) and was absent in wildtype, *Ybx2* ^+/-^ and *Ybx3* ^+/-^ mutants (Lanes 1–3).
(TIF)

S5 Fig Schematic of single and compound heterozygote testis RNA-sequencing analysis. Whole adult testes from duplicate wildtype, *Ybx2* ^*+/-*^, *Ybx3* ^*+/-*^, and compound *Ybx2/3* heterozygotes were collected for polysome fractionation. Prior to fractionation, a portion of the lysate was reserved for total RNA isolation. After fractionation, fractions corresponding to the mRNP or polysome were pooled and RNA isolated. Prior to library production, Spike-in RNAs were added to each RNA pool to facilitate library complexity normalization. One hundred base pair paired end sequencing was followed by alignment and gene-level abundance estimation. Estimates were normalized using Spike-in RNAs. Downstream analyses are discussed in detail in the text and materials and methods.
(TIF)

S6 Fig Comparison of biological duplicates used for RNA-sequencing analysis. Correlation of abundance estimates for each duplicate pair of samples demonstrating high levels of agreement in all but one (*Ybx3* ^*+/-*^ polysome) pair. R^2^ values indicated.
(TIF)

S7 Fig Gene expression differences in single and compound heterozygote testes. (A) Fraction of detected genes with altered expression as determined by EBSeq in compound heterozygotes demonstrating minimal impact on gene expression. Comparison of gene expression impact in single and compound heterozygotes. Genes with altered expression in compound heterozygotes were most often mis-regulated in one but not both of the single heterozygotes suggesting the gene expression profile in compound heterozygotes is a summation of single heterozygote impacts. (B) Cell-type expression of genes with altered expression in compound heterozygotes demonstrates impacted genes are predominantly expressed in the cell populations expressing YBX2 and YBX3.
(TIF)

S1 Table Primers for *Ybx2* and *Ybx3* genotyping. (DOCX)
