# Supplementary figures and images for: Compound Heterozygosity for Y Box Proteins Causes Sterility Due to Loss of Translational Repression
Source: PLoS Genet. 2015 Dec 8;11(12):e1005690. doi: 10.1371/journal.pgen.1005690 (PMC4672889; doi:10.1371/journal.pgen.1005690)

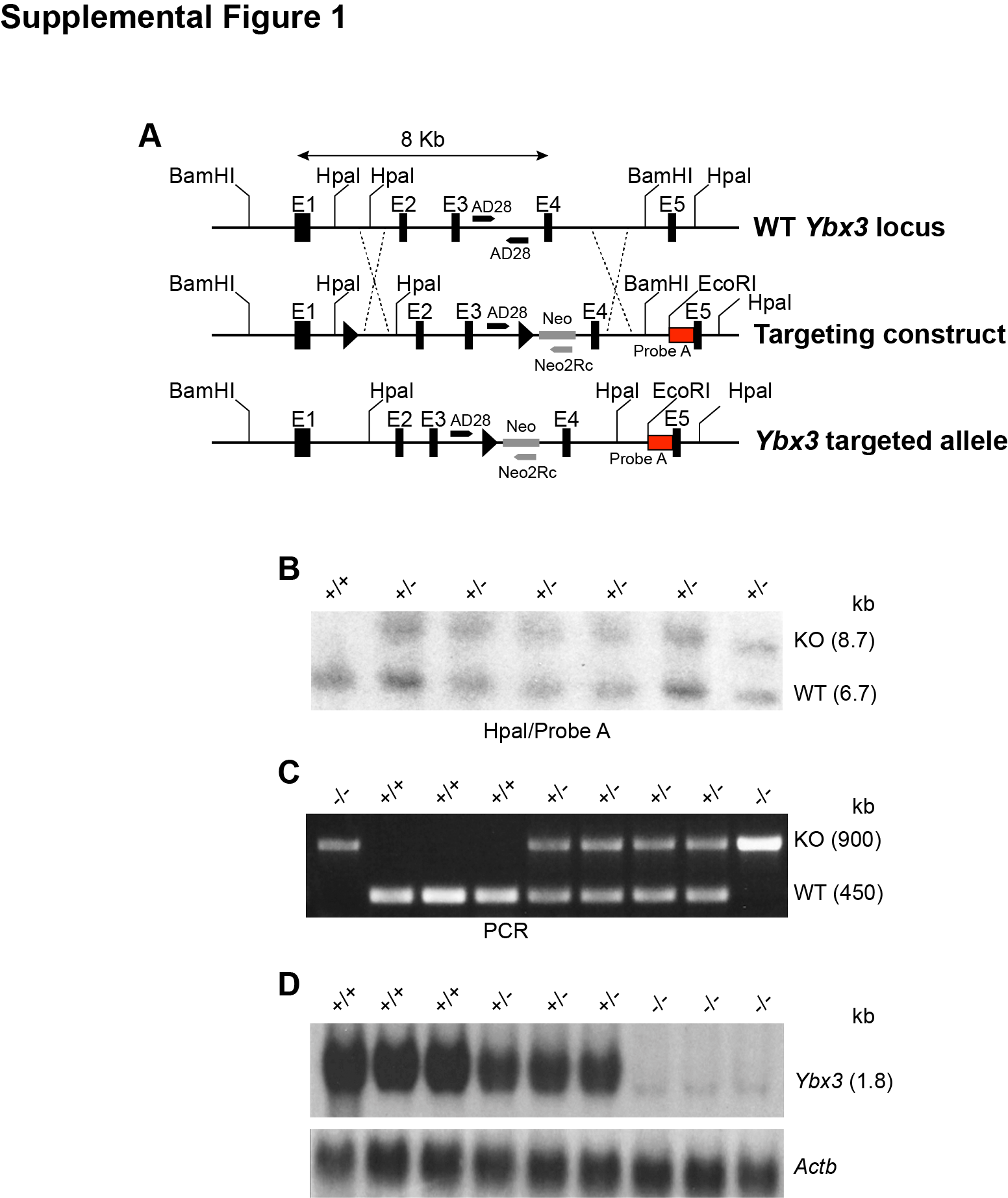

Supplement: S1 Fig — (A) Schematic representation of the Ybx3 gene-targeting construct and the targeted Ybx3 locus is shown. (B) Southern blot analysis the targeted ES +/- cells revealed homologous recombination of Ybx3 gene. The wildtype allele (6.7 kb) and the Ybx3-targeted allele (8.7 kb) were observed in the ES cells. An unexpected recombination event resulted in insertion of a single loxP site and the neomycin selection cassette upstream of exon 4, leading to disruption of the endogenous Ybx3 locus. (C) Genotyping using gene-specific primers detected a 900bp and a 450bp product in the heterozygous mutant, a 900bp product in the Ybx3 -/- mutant and a 450bp product in wild type mice. (D) Northern blot analysis demonstrates a significant decrease in Ybx3 transcript (1.8 kb) in Ybx3 -/- mutant testis compared to wild type or heterozygous testis. Actb was used as a loading control. (TIF) [file pgen.1005690.s001.tif]

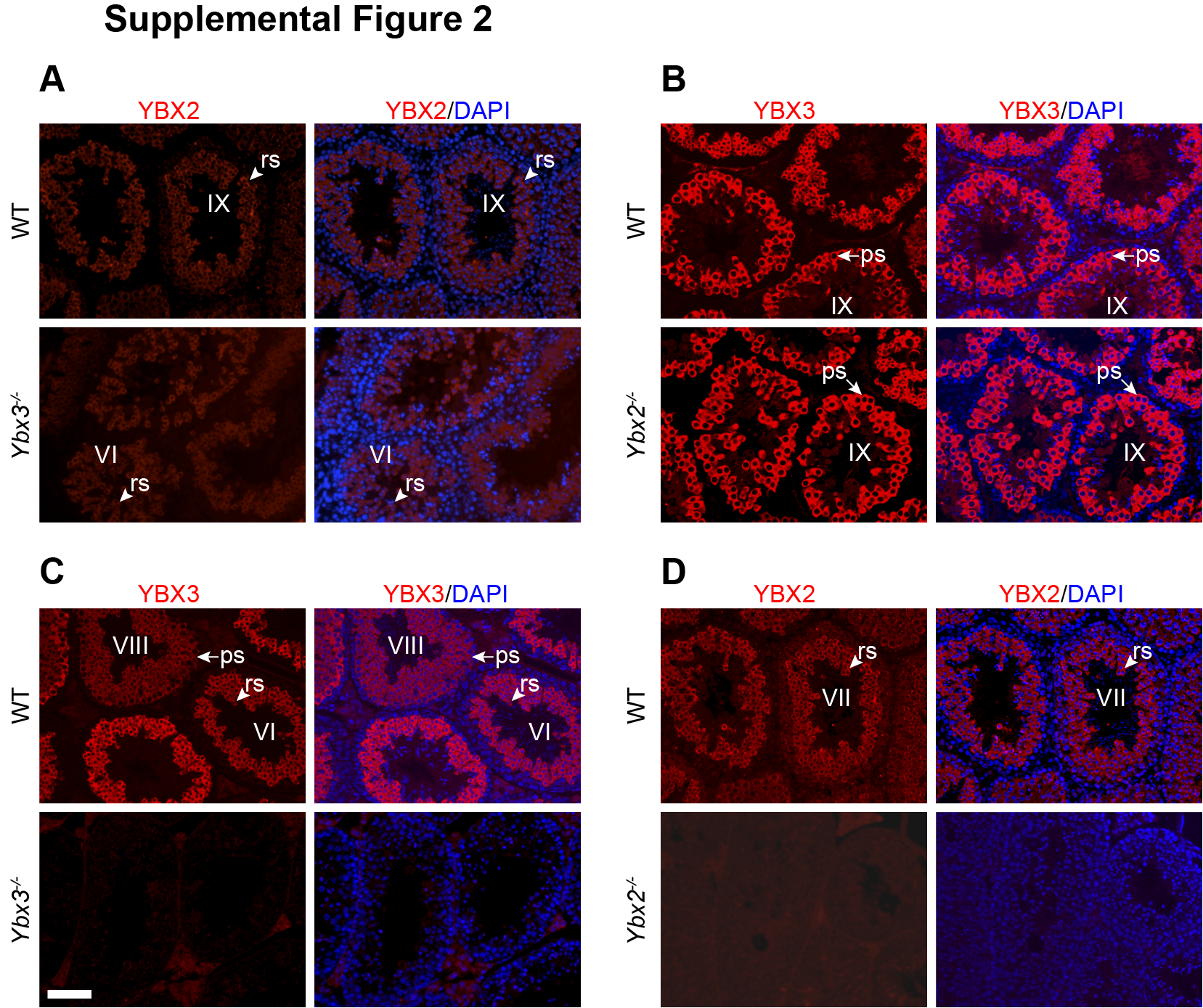

Supplement: S2 Fig — (A) YBX2 protein was expressed in round spermatids (rs, arrowhead) at stage VI in Ybx3 -/- mutant adult testis. (B) YBX3 protein was detected in the pachytene spermatocytes (ps, arrow) and round spermatids (rs, arrowhead) in Ybx2 -/- mutant and wildtype testis. (C) Immunofluorescence staining of adult testis sections with an N-terminal YBX3 antibody reveals a complete absence of YBX3 protein in Ybx3 -/- mutant testis. Cytoplasmic staining of YBX3 in pachytene spermatocytes (ps) of wildtype mice is shown (arrow). YBX3 staining in the cytoplasm of the round spermatids (rs) is shown (arrowhead). (D) Complete absence of YBX2 protein in Ybx2 -/- mutant testis relative to the wildtype testis. Scale bar = 100 μm. (TIF) [file pgen.1005690.s002.tif]

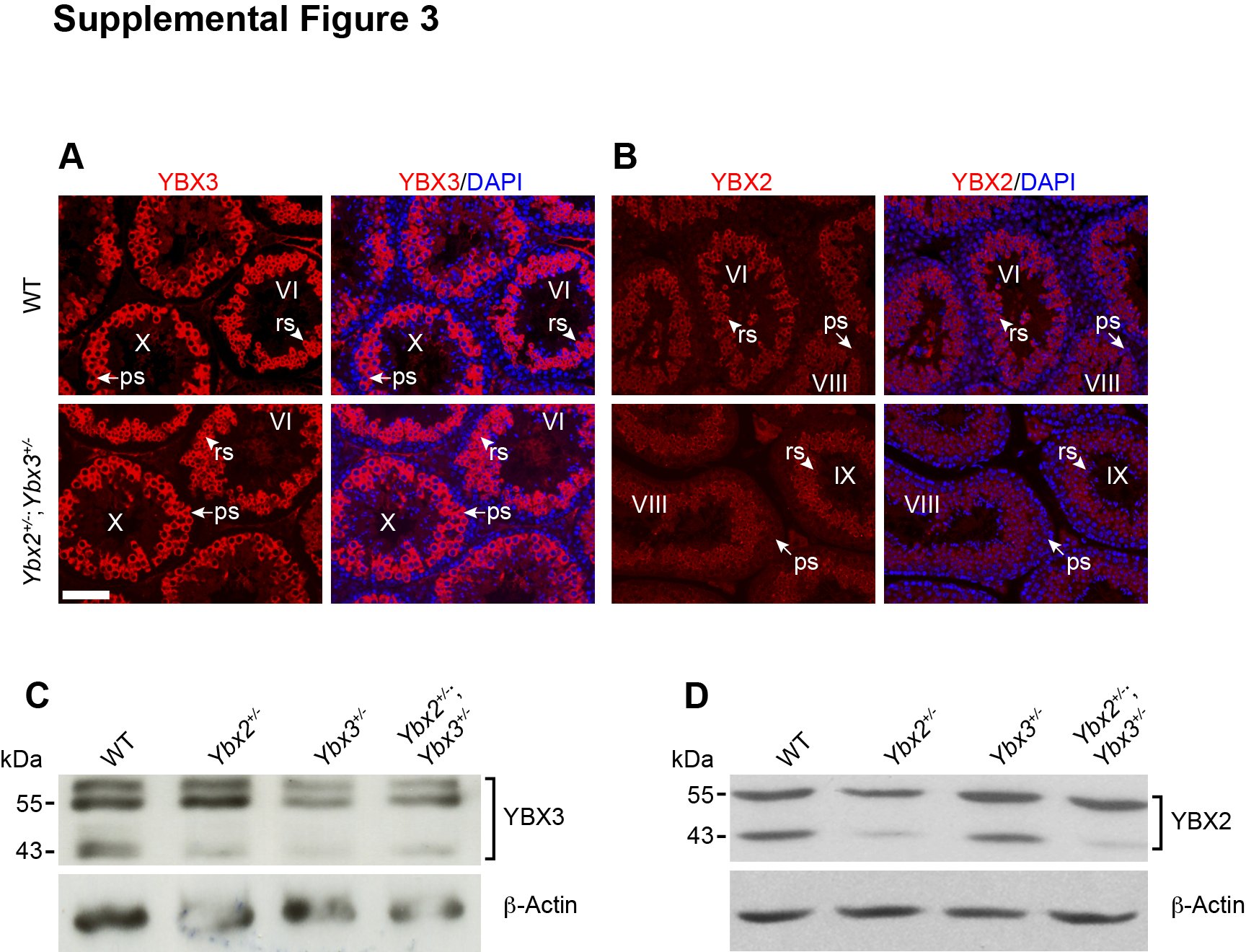

Supplement: S3 Fig — (A) YBX3 is expressed in late stage pachytene spermatocytes (ps) (arrow) and early to mid-stage round spermatids (rs) (arrowhead) in compound heterozygous mutant testis. (B) YBX2 is expressed in mid-stage pachytene spermatocytes (ps) (arrow) and early to mid-stage round spermatids (rs) (arrowhead) in compound heterozygous mutant testis. (C) Western blot analysis of YBX3 or YBX2 (D) proteins demonstrates a decrease in expression of these proteins in compound heterozygous mutant testis. Scale bar = 100 μm. (TIF) [file pgen.1005690.s003.tif]

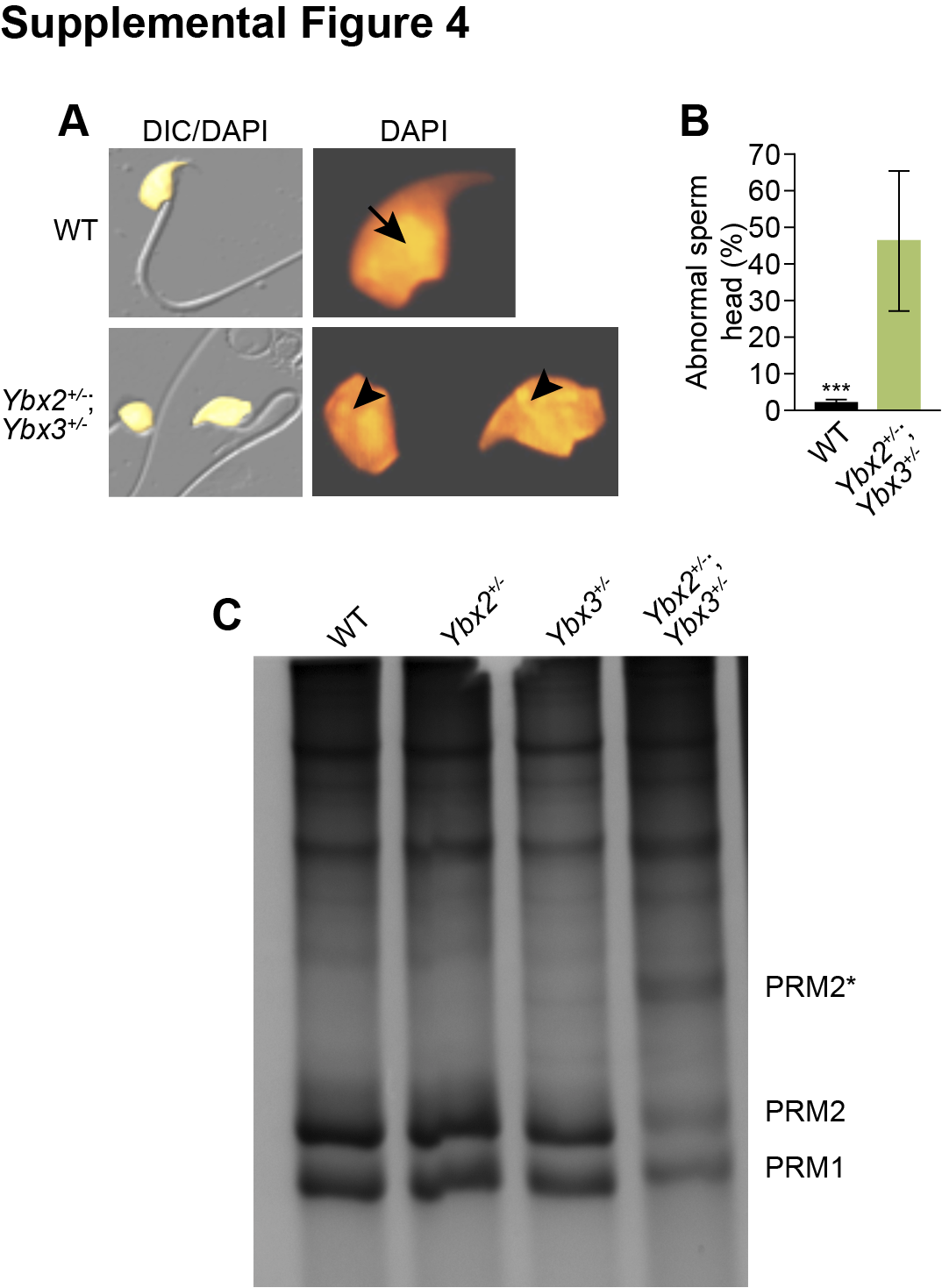

Supplement: S4 Fig — (A) Differential interference contrast (DIC) and isosurface rendering of DAPI staining revealed abnormalities in sperm head and tail morphology in compound heterozygous mutant mice (left-hand images). Serial images of individual sperm heads were further resolved using a deconvolution algorithm and then rendered using a red-to-yellow heat map and a fixed color isosurface (right-hand images). The 3D reconstruction of the sperm head (isosurface) revealed abnormal chromatin organization in compound heterozygous mutant mice. (B) Abnormal sperm head morphology was quantified and data expressed as a percentage ± SD. There was a significant increase in the abnormal sperm head morphology in compound heterozygous mutant relative to wildtype mice. N = 3. p < 0.001. (C) Nucleoproteins extracted from 1 x 10^6^ sonication-resistant sperm nuclei was fractionated on 15% Acid Urea PAGE, stained with Napthol blue, imaged using a G-Box Chemi-XT4 Synoptics camera and analyzed by GeneSys software. The positions of protamine 1 (PRM1), mature protamine 2 (PRM2) and the unprocessed PRM2 precursor (PRM2*) are listed. PRM2* was detected only in compound heterozygous mutants (Lane 4) and was absent in wildtype, Ybx2 +/- and Ybx3 +/- mutants (Lanes 1–3). (TIF) [file pgen.1005690.s004.tif]

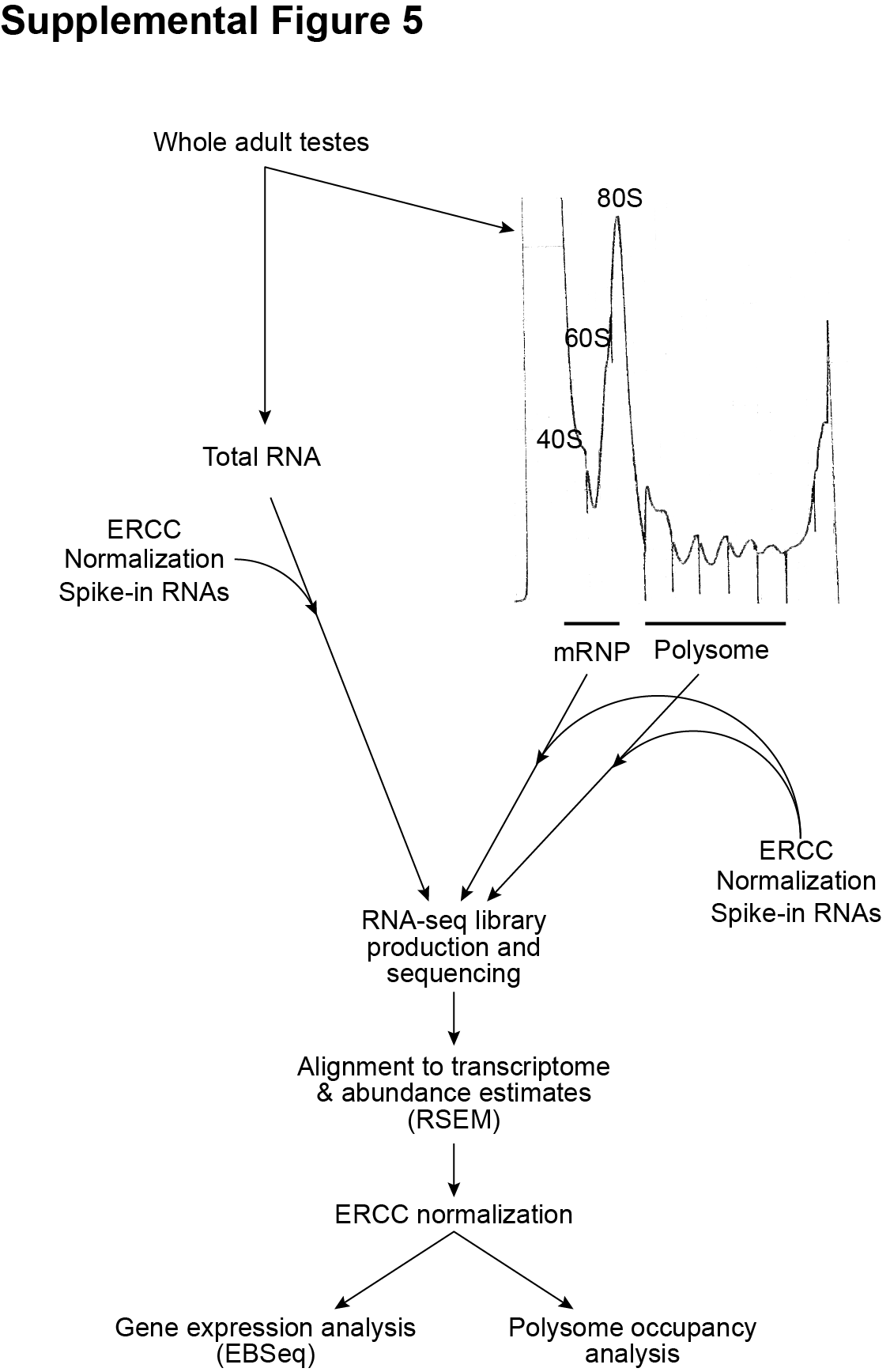

Supplement: S5 Fig — Whole adult testes from duplicate wildtype, Ybx2 +/-, Ybx3 +/-, and compound Ybx2/3 heterozygotes were collected for polysome fractionation. Prior to fractionation, a portion of the lysate was reserved for total RNA isolation. After fractionation, fractions corresponding to the mRNP or polysome were pooled and RNA isolated. Prior to library production, Spike-in RNAs were added to each RNA pool to facilitate library complexity normalization. One hundred base pair paired end sequencing was followed by alignment and gene-level abundance estimation. Estimates were normalized using Spike-in RNAs. Downstream analyses are discussed in detail in the text and materials and methods. (TIF) [file pgen.1005690.s005.tif]

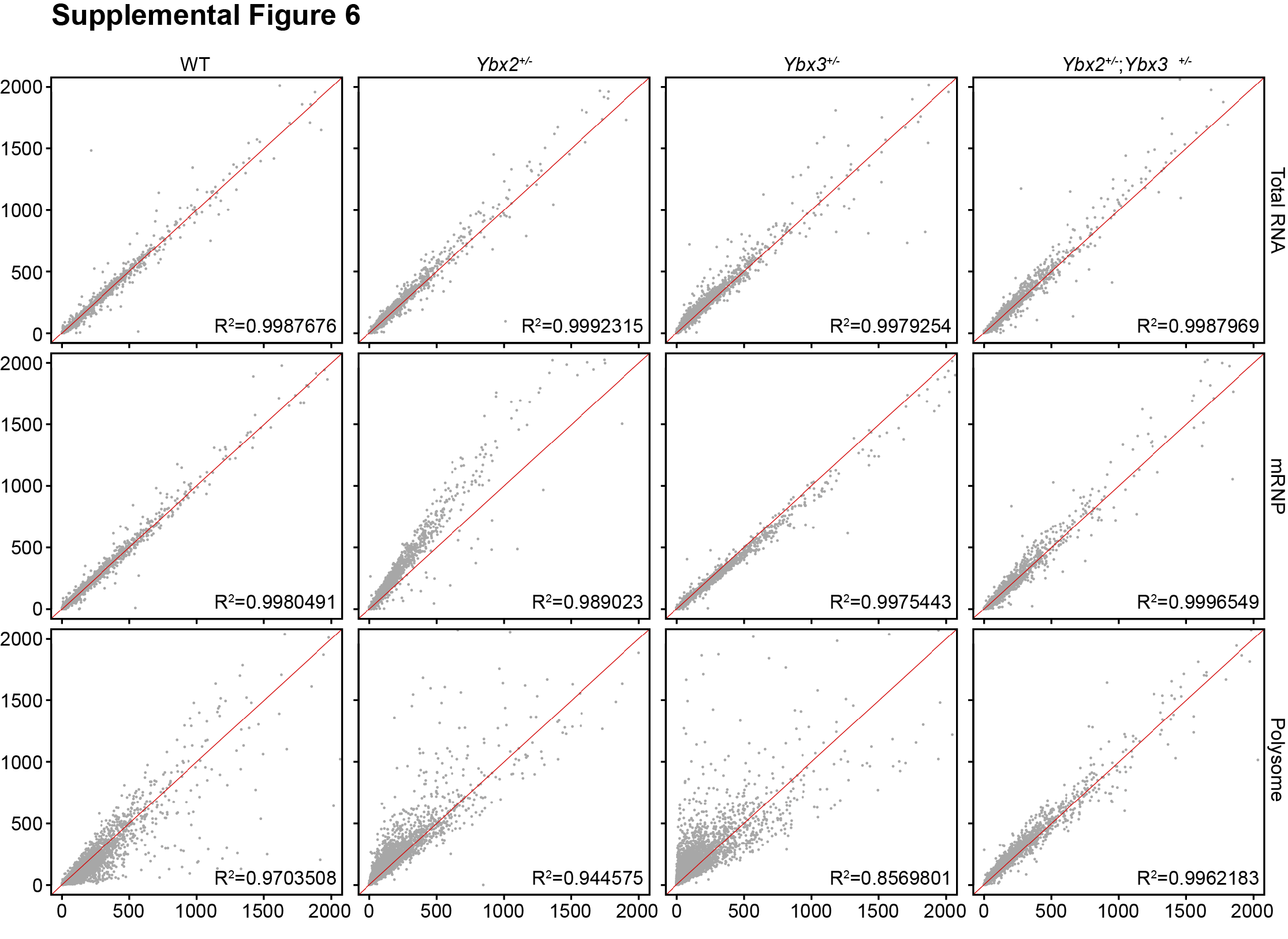

Supplement: S6 Fig — Correlation of abundance estimates for each duplicate pair of samples demonstrating high levels of agreement in all but one (Ybx3 +/- polysome) pair. R2 values indicated. (TIF) [file pgen.1005690.s006.tif]

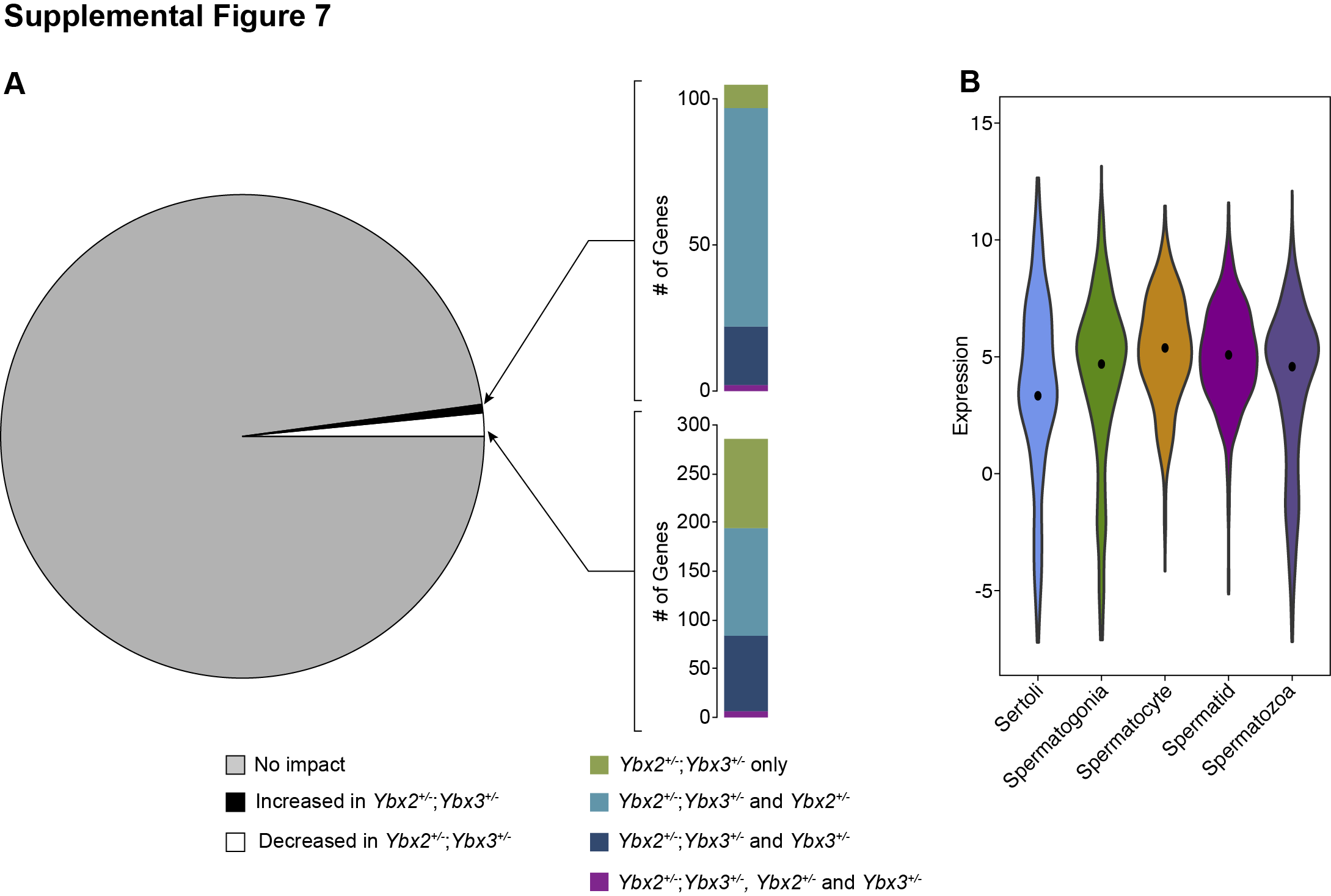

Supplement: S7 Fig — (A) Fraction of detected genes with altered expression as determined by EBSeq in compound heterozygotes demonstrating minimal impact on gene expression. Comparison of gene expression impact in single and compound heterozygotes. Genes with altered expression in compound heterozygotes were most often mis-regulated in one but not both of the single heterozygotes suggesting the gene expression profile in compound heterozygotes is a summation of single heterozygote impacts. (B) Cell-type expression of genes with altered expression in compound heterozygotes demonstrates impacted genes are predominantly expressed in the cell populations expressing YBX2 and YBX3. (TIF) [file pgen.1005690.s007.tif]
