# Supplementary material for: Compound Heterozygosity for Y Box Proteins Causes Sterility Due to Loss of Translational Repression
Source: PLoS Genet. 2015 Dec 8;11(12):e1005690. doi: 10.1371/journal.pgen.1005690 (PMC4672889; doi:10.1371/journal.pgen.1005690)
Supplement: S1 Table — (DOCX) [file pgen.1005690.s008.docx]

| Gene | Primers (5’ to 3’) | Amplicon size (bp) |
| --- | --- | --- |
| *Ybx2* | M2In3  (Forward):CGGATTGTTGTTTAACTTGTAAATG  M2Ex4R (Reverse):TGCAGAAGAGGATGGGTTGTTAAGG  HPRT-3225F  (Internal):CGGATTGTTGTTTAAGTTGTAAATG | Wt: 620  Mut: 520 |
| *Ybx3* | AD28  (Forward):TAGAGGAAGGCTGGATTACA  AD30  (Reverse):GAAAACATGAGGTGGTGACT  Neo2 RC  (Internal):CAGGACATAGCGTTGGCTAC | Wt:341  Mut:900 |
